# Multidrug Resistant Invasive Nontyphoidal Salmonella Isolated from and Masquerading Healed Tubercular Constrictive Pericarditis and Study of Virulence Markers

**DOI:** 10.7759/cureus.1198

**Published:** 2017-04-26

**Authors:** Reetika Dawar, Anoop Ganjoo, Firdaus Imdadi, Vasundhra Bhandari

**Affiliations:** 1 Microbiology, Indraprastha Apollo Hospital, New Delhi; 2 Department of Cardiothoracic Vascular Surgery, Indraprastha Apollo Hospital, New Delhi; 3 Dst-inspire Faculty, National Institute of Animal Biotechnology

**Keywords:** virulence gene profiling, infective pericarditis, maldi-tof, multidrug resistant, invasive nontyphoidal salmonella(ints)

## Abstract

Among the infectious causes of pericarditis are various bacteria, viruses, fungal and parasitic infections. The course disease may progress to a chronic constrictive pattern especially with tubercular etiology. Non-typhoidal Salmonella has rarely been reported as a cause of pericarditis. We describe here a case from which the pericardial fluid from an old case of tubercular pericarditis sent for culture to microbiology laboratory grew a *Salmonella typhimurium*. We studied the antibiotic resistance pattern, phage type and virulence factors playing a role in the invasive nature of the pathogen since no such study from pericardial fluid was found in the literature.

The isolate was sensitive only to cephalosporins and it was untypable. It showed amplification for five fimbrial operons, three colonization factors, and other genes (pef operon), gog B(Gifsy-1 encoded effector), sseI (Gifsy-2 encoded effector), sodC ( the periplasmic [copper and zinc Cu, Zn ]-superoxide dismutase) & sopE (a guanine nucleotide exchange factors).

The present case highlights the need for early detection of the exact causative agent and serovar in management, the likelihood of a different etiological agent other than the original to be kept in mind for timely management and the highly resistant pattern of non-typhoidal Salmonella (NTS) limiting the therapeutic options as in our case to only cephalosporins. The genes encoded from the NTS might be required for invasive cardiac manifestation in humans.

## Introduction

Salmonellosis is an endemic disease in India. The most common clinical presentation is as typhoid fever. Nontyphoidal Salmonella (NTS) commonly cause self-limiting gastroenteritis, and bacteremia occurs in only five percent of patients, but severe extraintestinal focal infections including septic aortitis, meningitis, pneumonia, septic arthritis, osteomyelitis or cholangitis have been reported earlier [1]. With the widespread use of antibiotics, it is rare for bacterial pericarditis to progress to a chronic course except with etiological agents like Mycobacterium tuberculosis. Pericarditis was first reported in association with typhoid fever in 1844.

Over the years, there have been an increasing report of multidrug resistance in Salmonella and more so in NTS. The key to recovery is effective antibiotic usage.

The Centre for Disease Control, Atlanta recognizes two species which are divided into seven subspecies: S.enterica (six subspecies) and S.bongori (one subspecies). Different methodologies for Salmonella enterica subspp enterica subtyping exist, serotyping being the most popular method. Other methods include antibody microarrays and phage typing. Molecular typing techniques which have been employed in various centers are pulse-field gel electrophoresis, deoxyribonucleic acid (DNA) base serotyping, multiplexed ligase chain reaction assays, polymerase chain reaction (PCR), subtyping based on whole -genome comparisons, multilocus sequence typing, single-nucleotide polymorphism (SNP) typing, multilocus variable number tandem repeat analysis and molecular typing with composite microarrays. Whole -cell matrix desorption ionization - a time of flight mass spectrometry (MALDI-TOF MS) is a direct bacterial profiling method based on detection of surrogate marker proteins. We have used a combination of methodologies available to us even though it is a single strain, and phage typing is not essential here as we do not expect many cases to be reported with such presentation and thus we studied the virulence factors and phage type to have a database for Salmonella Typhimurium.

S .typhimurium is an intracellular pathogens, and different sets of specific virulence factors are responsible for its internalization, survival, replication and also act as effectors targeting various stages in the cellular processes of infection, invasion and intracellular infection. Salmonella can actively invade both phagocytic and nonphagocytic cells using a type III secretory system. Also, a group of effector proteins (SipA, SipC, SopB, SopD, SopE ) induce rearrangements in the actin cytoskeleton resulting in internalization of the bacteria. Other factors like fimbriae, flagella may play a role.

We report here a case of nontyphoidal Salmonella pericarditis in a patient with past history of tubercular constrictive pericarditis and we also looked for the probable virulence factors.

## Case presentation

A 46-year-old female who was a resident of Nigeria was admitted to our hospital with complaints of breathlessness on minimal exertion and swelling of her face and legs since last seven months. She was a known case of tubercular constrictive pericarditis diagnosed about 15 years back and treated with a full course of antitubercular therapy. She was on medical follow-up in her country and had minimal to no symptoms for several years. However, about seven months back she was hospitalized for abdominal pain of seven days duration. The hospital records of that time mentioned heart failure and advice for pericardiectomy and she was referred to our hospital for specialized care. On admission here, she appeared to be sick looking with an irregular pulse rate of 96/min, raised jugular venous pressure (JVP), bilateral pedal edema and muffled heart sounds. Routine blood tests were unremarkable. Sickle cell trait was negative. Anti- hepatitis C virus (HCV) antibodies were reactive. Ultrasound abdomen did not reveal any cirrhosis. The patient was human immunodeficiency virus (HIV) negative. Two- dimension echocardiogram (2-D ECHO) showed evidence of calcified constrictive pericarditis. Cardiac catheterization showed chamber pressures consistent with constriction. Coronary arteries were normal.

The patient was taken up for pericardiectomy and during operation, the pericardium was found to be heavily calcified. While dissecting along the inferior surface, a small pocket of creamy white fluid was encountered. Excised pericardium was sent for histopathology and pericardial fluid to microbiology for aerobic culture and sensitivity, Ziehl Neelsen’s stain (ZN) and culture for mycobacteria.

The patient was empirically put on amoxicillin plus clavulanate (amoxicillin 875 mg plus clavulanic acid 125 mg) twice a day & cefuroxime 500 mg twice a day till culture results were available. The aerobic culture put on Columbia blood agar and MacConkey agar showed pure growth of non-lactose fermenting colonies after 24 hours of incubation at 37o C. These were oxidase negative, motile, citrate positive, triple sugar iron (TSI) agar showed the alkaline/acid reaction with hydrogen sulfide (H2S) and gas production. Lysine decarboxylase was negative. The isolate was processed for identification of Vitek -2 Compact Systems and Vitek -MS (BioMerieux, Marcy l'Etoile, France). Serotyping was done using Salmonella antisera. The lactose non-fermenting isolate was identified as Salmonella group on Vitek -2 Compact Systems. The strain showed agglutination with Salmonella antisera polyvalent O, Salmonella O 4 antiserum and Salmonella antiserum H-i (Denka Seiken Co.Ltd, Japan). On Vitek –MS (which is based on MALDI-TOF technology) it was identified as Salmonella group (99.9% confidence value) and further as Salmonella enterica spp enterica by analyzing the mass data in Spectral Archive And Microbial Identification System (SARAMIS software, AnagnosTec Germany) and processed with baseline correction, Gaussian smoothing and peak finding. The isolate was thus identified as Salmonella enterica subspp enterica serovar Typhimurium based on the above techniques.

Antibiotic susceptibility testing was performed as per the Clinical and Laboratory Standards Institute (CLSI) guidelines by Kirby-Bauer method and also put in Vitek 2 compact (BioMerieux, France) using antimicrobial susceptibility testing (AST-N280) card. The isolate was sensitive to ceftriaxone, cefixime and resistant to nalidixic acid, cotrimoxazole, chloramphenicol, ampicillin, and quinolones. The isolate was sent for phage typing to National Salmonella Phage Typing Centre, Lady Hardinge Medical College, New Delhi, keeping in view the high resistance pattern even though phage typing is done mostly for epidemiological investigation of outbreaks. The isolate was reported as ‘untypable’.

To further understand the genetic profile of the invasive nontyphoidal serovar (i NTS) and know the virulence gene content of the isolate, polymerase chain reaction (PCR) was performed. Amplified PCR products were separated by agarose gel electrophoresis and stained with ethidium bromide to assess the presence of specific band size of each primer set (Table 1) and gels were photographed under gel documentation system (Syngene).

**Table 1:** List of primers List of primers with forward and reverse primer sequences

S.No	Primer name	Forward Primer	Reverse Primer
	sopE	CATAGCGCCTTTTCTTCAGG	ATGCCTGCTGATGTTGATTG
	pefA	TAAGCCACTGCGAAAGATGC	GCGTGAACTCCAAAAACCCG
	sodC	ATGACACCACAGGCAAAACG	AGATGAACGATGCCCTGTCC
	sseI	CGCCATCATCAGTAACCGCC	CTGCTGACCACATCCTCCC
	gogB	ACGAGGCGACATCAAACCTT	GACCGTTCCCTCAATCGTGT
	tcfA	TCGCTATGTTTGCATGTGGT	TTCAGGAACAGCCTCGAAGT
	hlyE	GCGTGATTGAAGGGAAATTG	CGAAAAGCGTCTTCTTACCG
	cdtB	CACTCGGCTATTGATGTTGG	ATTTGCGTGGGTTCTGTAGG
	Bcf	ATGAAAAAGCCTGTACTAGCA	TCAGGAATAAACCATGCTAAA
	Csg	ATGCCCCGCGCACAAAGTTAC	CAGGATTCTGGCGGTACTGA
	stbD	ATGCTTTTTAGTTTCCGAACG	TTACTGAAAACTAACCACTGCC
	sthA	ATGTTTAATAAGAAAATAATC	TTACTGATACGAAACGGTATA
	stiA	ATGAAACTCTCCTTAAAAACA	TCAGTTATATTGTAGATAGAA
	misL	ATGCCAACTCCCCAAAATTAC	CGTGACGCATCCCGCATGGCG
	bapA	ATGCGTCTACTCGCCGTGGTT	ATCTACAGGATTACTGCTACC
	sinH	GTTGTCGCCGATAAAAGTGAC	AGGCTGACGCCACCAATGAAC
	taiA	TGAACCCAAACCTGTTGTGA	GCAACATAAATTCGCTAATCTC

**Figure 1:**
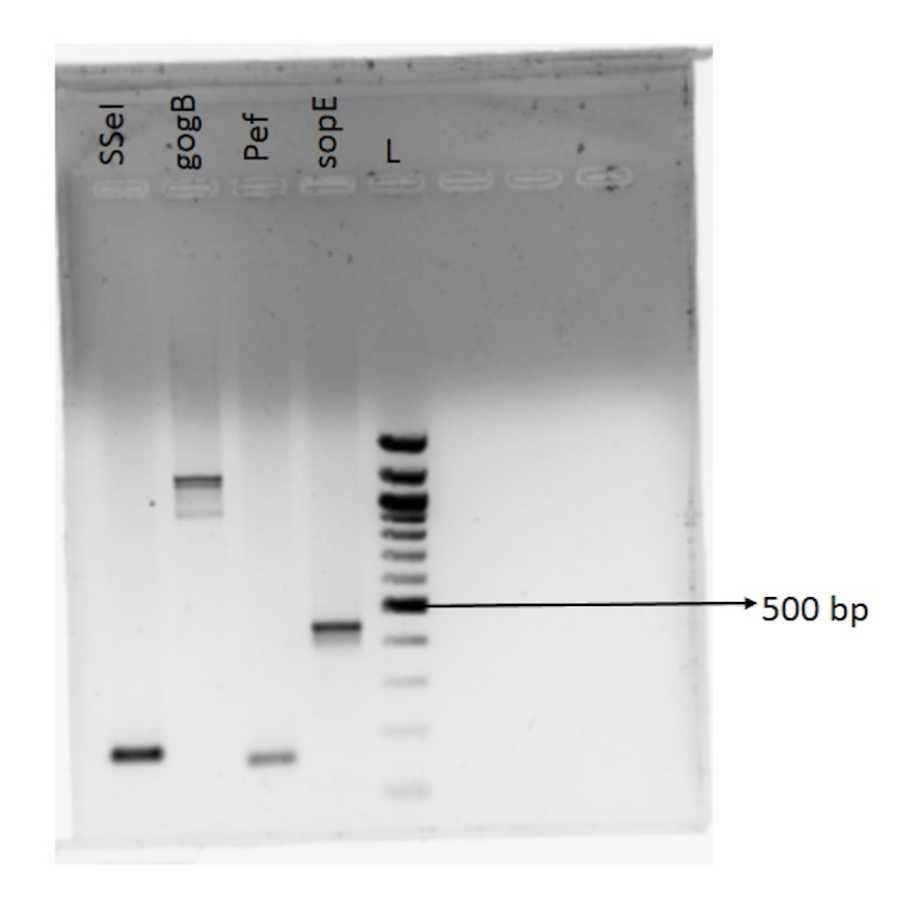
Amplified PCR products Bands separated by agarose gel electrophoresis

**Figure 2:**
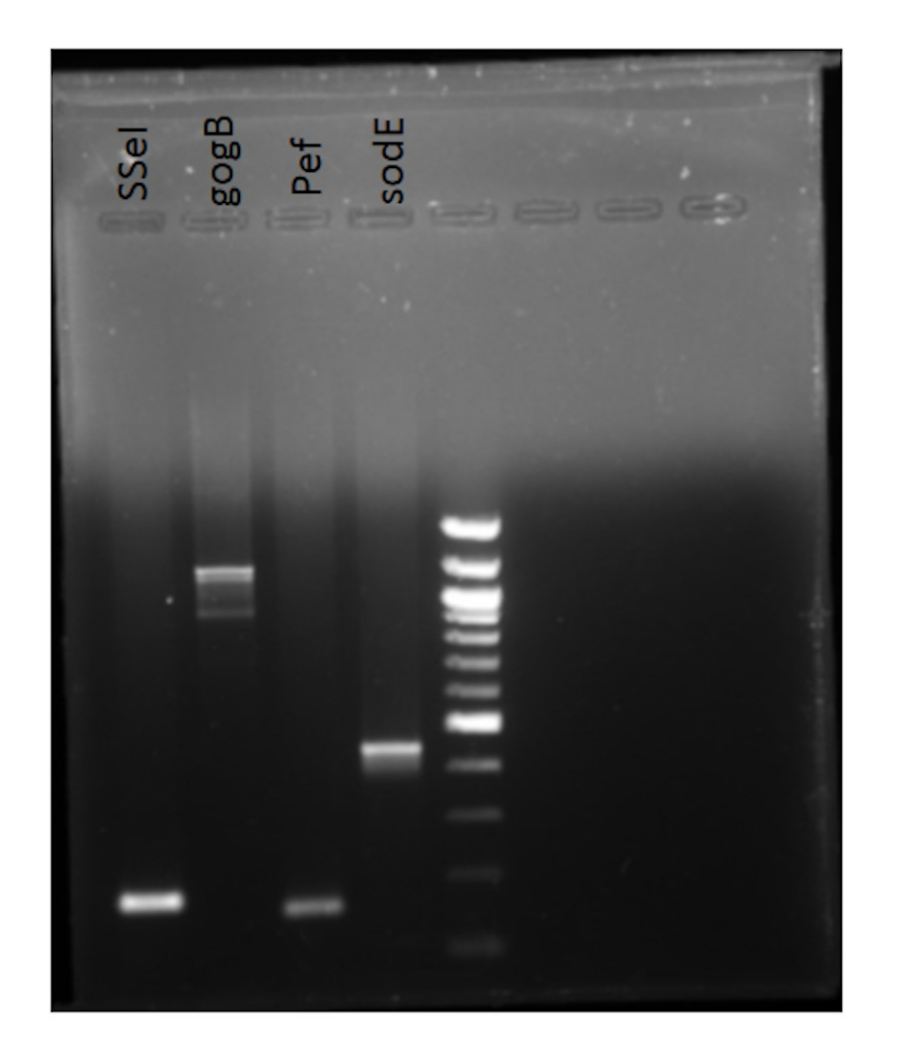
Amplified PCR products Bands separated by agarose gel electrophoresis

**Figure 3:**
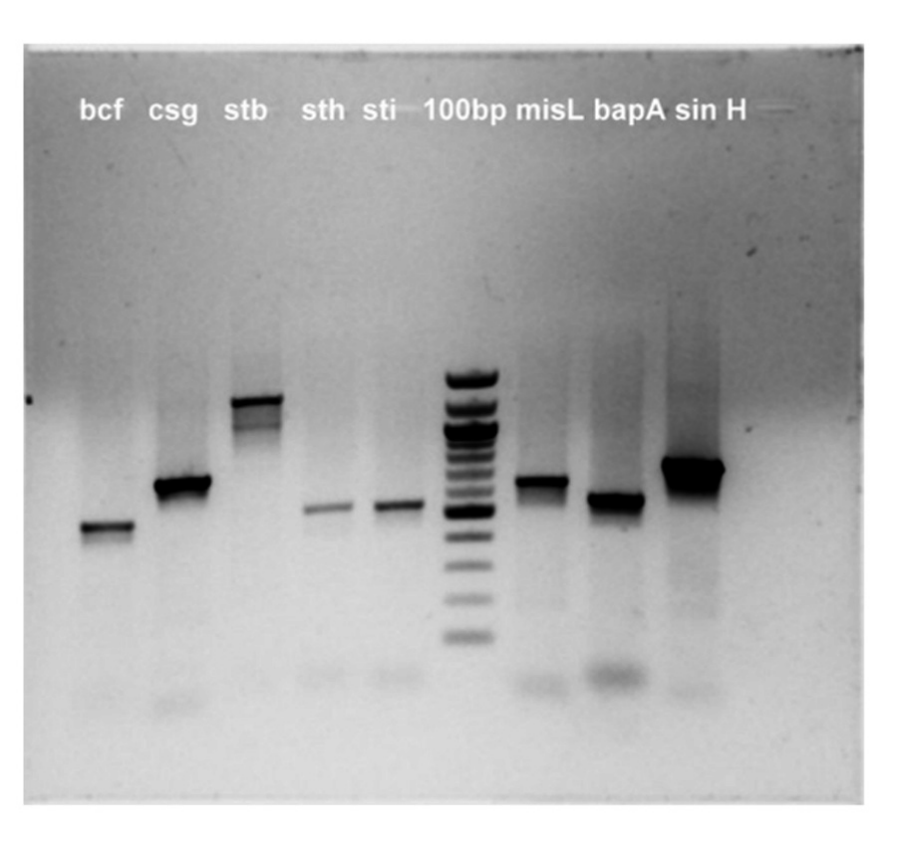
Amplified PCR products Bands separated by agarose gel electrophoresis

The strain showed amplification for the five fimbrial operons (bcf, csg, stb, sth, sti), sseI, gogB, pef, sopE ), three colonization factors (misL, bapA, sinH) and other genes- pef operon, gog B, sseI, sodC, sopE (Figures 1 -3). No amplification was seen for tcf A, CdtB, hlyE, taiA thus indicating that their function may not be required for pericardial invasion for Salmonella typhimurium.

Histopathology report of the excised pericardium showed chronic inflammation with fibrosis and calcification. No granulomatous inflammation was evident. ZN stain and GeneXpert assay were negative for Mycobacteria. Mycobacterial culture processed in mycobacteria growth indicator tube (MGIT 960) (Becton-Dikinson Ltd, Franklin Lakes, New Jersey, United States) showed no growth till six weeks of incubation.

The patient was treated with ceftriaxone 2 gms intravenously twice a day for two weeks followed by Cefixime 400 mg once a day for four weeks. The patient has not shown any residual bloodstream infection on repeat blood cultures and no relapses till now.

## Discussion

NTS pericarditis is uncommon. A literature review of 23 cases of NTS pericarditis between 1936- 1986 revealed four patients with pericarditis caused by Salmonella paratyphi. Paratyphoid fever with pericarditis was reported in an 11-year-old male in 2002 [2]. The documented host risk factors for non-typhoid Salmonella infections are children and infants less than three years old and endovascular infections in adults aged > 50 years, malaria, anemia, malnutrition, HIV infection, sickle cell disease and Schistosomiasis [3]. Our patient had none of these predisposing conditions. The incidence of bacterial pericarditis is five to 10 per 100,000 patients in Western countries and tuberculous pericarditis is less than four (but much more in Africa and South America) [4].

In a study on infective pericarditis in children in Nigeria, 31% were due to Mycobacterium tuberculosis and three percent due to Salmonella typhi. Both causes can lead to a constrictiv pattern [4]. The course can worsen requiring, besides medical treatment surgical intervention too. A retrospective study of children with tuberculous constrictive pericarditis from Africa showed that five out of 12 patients required pericardiectomy. Thus a quick identification of the NTS is critical in the management of the patient. Most Sub-Saharan African sites have consistently reported S. Typhimurium and S. enteritidis as the commonest invasive non-typhoidal Salmonella (iNTS) isolates.

MALDI-TOF MS is a direct bacterial profiling method based on detection of surrogate marker proteins and thus is quick in identification which can greatly help in early effective management.

Comparative genomic hybridization using a Salmonella enterica microarray revealed a core of 3233 genes present in all of the iNTS strains, which include the Salmonella pathogenicity islands 1-5, 9, 13, 14; five fimbrial operons (bcf, csg, stb, sth, sti); three colonization factors (misL, bapA, sinH); and the invasion gene, pagN. In the iNTS variable genome, Suez J, et al. identified in addition 16 novel genomic islets; various NTS virulence factors and six typhoid-associated virulence genes (tcfA, cdtB, hlyE, taiA, STY1413, STY1360) [5].

The virulence factors which might have played a role in invasion or causing pericarditis were found to be the five fimbrial operons (bcf, csg, stb, sth, sti), three colonization factors (misL, bapA, sinH) and other genes- pef operon, gog B, sseI, sodC, sopE; although this cannot be conclusive based on one single case report.

Pef operon encoded for plasmid-encoded fimbriae that mediate adhesion of Salmonella typhimurium to the small intestine of mice was present in our strain [6]. SopE was shown to be absent in 4/5 th of the blood isolates of serovar Typhimurium suggesting that it is non-essential for invasive salmonellosis in humans in an earlier study [5]. However, our strain showed the presence of this prophage encoded effector. No amplification was seen for tcf A, CdtB, hlyE & taiA for our isolate thus indicating that their function may not be required for pericardial invasion for Salmonella typhimurium.

Over the years the prevalence of multidrug resistance among Salmonella and resistance to clinically important antimicrobial agents such as fluoroquinolones has also emerged. Antimicrobial resistance of NTS to Trimethoprim-sulfamethoxazole, ampicillin and chloramphenicol is documented in Sub-Saharan Africa [4]. Antibiotic resistance pattern of NTS varies among different serovars. For example, there is a worldwide increase in the incidence of the multidrug-resistant serovar Typhimurium; namely the emergence of definitive type (DT104), which was first recognized in the UK in 1984. It is resistant to ampicillin, chloramphenicol, streptomycin, sulphonamides and tetracycline with all these resistance genes located on the chromosome. Combination of phage types, pulsed-field gel electrophoresis (PFGE) profiles and patterns of antimicrobial resistance of multidrug-resistant strains of Salmonella typhimurium needs to be done for continued surveillance and to monitor the dissemination of multi-drug-resistant (MDR) strains.

A total of 283 Salmonella typhimurium strains isolated from 187 cases of human infections and 96 non-human sources from the state of Sao Paulo, Brazil were examined for antimicrobial susceptibility and the incidence of resistance was 38% and multiple resistance (to three or more antimicrobials) was 15%. All 43 multidrug-resistant strains (MDR) and 13 susceptible ones were characterized by phage typing and PFGE. Three were untypable (UT) and one untypable phage type was resistance to ampicillin, nalidixic acid and Streptomycin [7].

In a report from Lagos, Nigeria of Salmonella-associated community gastroenteritis out of nineteen Salmonella typhimurium including three sporadic isolates, the three sporadic strains was untypable [8]. All the three were resistant to ampicillin, tetracycline, chloramphenicol and one were resistant in addition to sulfamethoxazole-trimethoprim.

The isolate in our case report was in addition resistant to nalidixic acid and cotrimoxazole limiting the use of these agents for the management. Third-generation cehalosporins have become the treatment of choice for pericarditis caused by ampicillin-resistant strains [9]. The good clinical response has been reported after treatment with ceftriaxone alone in ampicillin resistant non-typhoidal Salmonella bacteremia complicated with pericarditis [10]. Treatment in such case should be continued for a minimum of six weeks if surgical intervention is successful. Some consultants prescribe few months of suppressive therapy even when the patient is doing well.

## Conclusions

We report a rare case of Salmonella Typhimurium pericarditis from India. The aim is to highlight the potential of these bacteria to cause pericarditis with an aggressive course and rapid deterioration, thickening, and constriction requiring surgery. We also emphasize the importance of sending a diagnostic tap of the pericardial effusion and thus the impact microbiology culture has in the early and right choice of antibiotics. The virulence factors namely five fimbrial operons (bcf, csg, stb, sth, sti), three colonization factors (misL, bapA, sinH) and other genes- pef operon,gog B,sseI,sodC, sopE might have played a role in pericarditis; though further studies are needed to conclude their role.

The likelihood of a different etiological agent other than the original one should be kept in mind every time when there is worsening of symptoms even in an old case of constrictive pericarditis. The previously damaged pericardium was susceptible to seeding during a transient bacteraemia. In our case, NTS worsened the pre-existing pericarditis and led to hasten the need for a pericardiectomy which otherwise might have been done at a later stage. This is to the best of our knowledge, the first case reported of NTS pericarditis in a previous case of tubercular pericarditis. We speculate that the healed tuberculous pericarditis might provide a nidus for Salmonella infection, though this may not be conclusive as this being the only known case.

In addition, the highly resistant pattern of NTS limited the therapeutic options as in our case to cephalosporins is the only option.
